# Impact of the Covid-19 pandemic on mental health of persons with disabilities: Insights from the 2021 Canadian Housing Survey

**DOI:** 10.1371/journal.pgph.0004728

**Published:** 2025-06-04

**Authors:** Sulemana Ansumah Saaka, Roger Antabe, Isaac Luginaah

**Affiliations:** 1 Department of Geography and Environment, Faculty of Social Science, University of Western Ontario, London, Ontario, Canada; 2 Department of Health and Society, University of Toronto Scarborough, Toronto, Ontario, Canada; PLOS: Public Library of Science, UNITED STATES OF AMERICA

## Abstract

Mental health (MH) remains a major public health concern in Canada and has been exacerbated by the unprecedented challenges of the COVID-19 pandemic and the associated restrictions on physical movement. While considerable work has been done on the impact of COVID-19 on the physical and MH of the general population, relatively less work has focused on the MH of persons with disabilities (PWDs). Although the COVID-19 containment measures including lockdowns, social distancing, quarantine, and closure of nonessential services were intended to reduce the direct risks of COVID-19, the socioeconomic consequences of those restrictions and the uncertainties surrounding the virus, inadvertently had adverse impact on the MH and well-being of Canadian residents, particularly, among already marginalized groups such as PWDs. Moreover, PWDs were identified as disproportionately vulnerable to the psychological impacts of the pandemic containment measures which compromised their overall Positive Mental Health (PMH): a state of well-being where individuals can realize their full potential, manage life’s stresses, work productively, and contribute to society. This study addresses the research gap by examining the effect of the pandemic on the MH of PWDs in Canada using a cross-sectional analysis of the 2021 Canadian Housing Survey (N = 15,626), a subset of people who reported disabilities. Logistic regression models were employed for this cross-sectional analysis. The results show that females (OR = 0.789; P < 0.001), those who experienced COVID-19 economic hardship (OR = 0.703; P < 0.001), and dwelling dissatisfaction (OR = 0.585; P < 0.001), significantly reported about 0.79, 0.70, and 0.59 times lower odds of positive Mental Health (PMH), respectively. On the other hand, those who had post-secondary educational attainment (OR = 1.210; P < 0.001), strong sense of community belonging (OR = 2.056; P < 0.001), and civic engagement with their communities (OR = 1.204; P < 0.001), were significantly associated with 1.21, 2.06, and 1.20 times higher odds of PMH, respectively. Additionally, immigration status, household type, the province of residence, and neighborhood-specific challenges such as race-based harassments, and drug use/dealings emerged as significant predictors of PMH. The findings underscore the positive impacts of empowering elements such as strong community ties on the MH of PWDs during public health crisis. Also, the findings prompt the pressing need for identifying and addressing the unique challenges of PWDs in Canada, particularly, the less educated and socioeconomically disadvantaged, as part of effort to foster PMH in the country. Overall, these findings suggest the need to prioritize and strengthen disability-inclusive MH programs for future public health crises.

## Introduction

Globally, mental health persists as a major public health concern, particularly, during and after the COVID-19 pandemic. Specifically in Canada, evidence suggests that the COVID-19 pandemic has exacerbated mental health (MH) problems among several groups. Even though declines in population MH were evident in Canada prior to emergence of the COVID-19 pandemic, estimates from Statistics Canada indicate this might have substantially worsened during the COVID-19 pandemic. For example, findings from the country’s Mental Health and Access to Care Survey suggest that there were more than 5 million people in Canada experiencing significant symptoms of mental illness as of 2022. The key contributory factor to this high figure is the impacts of the COVID-19 pandemic on population health and access to health services [1]. It is also reported by the Public Health Agency of Canada that aside from the direct impact of COVID-19 on the physical health of Canadians, the adverse effects on MH outcomes were remarkably high [2,3]. For instance, available evidence indicates a stark rise in MH challenges during the second wave of the pandemic in the fall of 2020—as the percentage of Canadian adults who screened positive for major depressive disorders during the pandemic was at least 2 times higher compared to the pre-pandemic era [4]. Consequently, fewer adults reported good self-rated MH and community belonging, marking a significant shift from the pre-pandemic era [5,6]. Against the backdrop of deteriorating MH, disabilities and vulnerabilities stemming from pre-existing health conditions, as well as Covid-induced economic hardships were among other factors that accentuated the risk of poor MH outcomes [7].

Prior to the COVID-19 pandemic, Persons with disabilities (PWDs) were already facing challenges in terms of mobility, social contact, access to healthcare services, housing suitability challenges, risk of loneliness, emotional distress, and poorer health-related quality of life when compared to people without disability [8]. These challenges were exacerbated during the pandemic [9]. For instance, globally, governments implemented various responses to the pandemic, including travel restrictions, lockdowns, social distancing, quarantine, and closure of nonessential services. Like many other countries, while these measures were intended to reduce the direct risks of COVID-19, the uncertainties surrounding the virus, and the socioeconomic consequences of those restrictions inadvertently affected the overall Positive Mental Health (PMH) and well-being of Canadian residents [10]. PMH, a state of well-being where individuals can realize their potential, manage life’s stresses, work productively, and contribute to their communities, involves not only the absence of mental illness but also traits like resilience, self-esteem, emotional regulation, and social connectivity [11]. Emotional, psychological, and social well-being are key components of PMH which fosters a sense of purpose and satisfaction [11]. Overall, PMH enables individuals to thrive and function optimally in their communities.

While the term “***disability”*** is often defined as a physical, mental, or sensory condition that limits a person’s ability to perform certain activities or interact with the world around them, the current study adopts the definition of the Social Model of Disability (SMD) [12,13] where ***disability*** is conceived not solely as a result of an individual’s physical or mental impairments, but also, a condition shaped by the social, cultural, political, and economic factors that affect the experiences of PWDs [14]. Important distinctions between *impairment* (i.e., a functional limitation caused by physical, mental, or sensory defect), and *disability* (i.e., a loss or limitation of opportunities to equally partake in the normal life of a community due to both physical and social barriers) are thus made by the SDM [13,15,16], situating ***disability*** squarely within society [15,17]. This definition thus holds the view that the individuals’ impairment is not the cause of their inability to fully function (i.e., disability), but rather, society’s failure to consider and or provide appropriate services that meet the needs of PWDs in social organizations. It therefore draws attention to the dynamic socio-environmental conditions in which disability is constructed, making PWDs more vulnerable to adversities in society. For instance, recent studies show that the effects of COVID-19 were particularly more acute among already marginalized groups in Canada, including PWDs [18] and those in substandard housing. According to the World Health Organization (WHO), PWDs are at 2 times higher risk of developing depressive conditions, asthma, diabetes, stroke, and obesity. PWDs experience these health inequities due to unfair social conditions including stigma, discrimination, poverty, and barriers faced in the healthcare system itself [19]. Also, they are reportedly 15 times more likely to experience difficulties than those without disabilities with regard to finding accessible and affordable transportation [19]. All these conditions were worsened by the COVID-19 restrictions on essential health services and daily movement, and consequently increased the vulnerability of PWDs to poorer mental health outcomes. Meanwhile, 27% of Canadians aged 15 years and above (i.e., 8 million people) had disability as of 2022, representing a 4.7% increase from 2017 [20]. Yet, existing literature on mental health ramifications of the COVID-19 pandemic in Canada has focused largely on the general population [21–24], limiting our understanding of the differential impact of the pandemic on PWDs who were already vulnerable to poor mental health prior to the pandemic. Thus, to fill this scholarly void and contribute to the broader literature, our study sought to examine the factors associated with mental health outcomes of PWDs during the COVID-19 pandemic, to present valuable insights for targeting and addressing the unique needs of PWDs in the event of future outbreaks.

### Theoretical context

This study employs both the social model of disability (SMD) [12,13] and the socio-ecological framework [25] to understand the unique MH challenges and experiences of PWDs during the COVID-19 pandemic. The SMD holds that disability is not solely a result of an individual’s physical or mental impairments but also a condition shaped by the social, cultural, political, and economic factors that affect the experiences of PWDs [14]. Important distinctions between *impairment* (i.e., a functional limitation caused by physical, mental, or sensory defect), and *disability* (i.e., a loss or limitation of opportunities to equally partake in the normal life of a community due to both physical and social barriers) have been made by the SDM [13,15,16]. The model situates disability squarely within society [15,17], implying that the individual’s impairment is not the cause of inability to fully function (i.e., disability), but rather, society’s failure to consider and or provide appropriate services that meet the needs of PWDs in social organizations. It, therefore, draws attention to the dynamic socio-environmental conditions in which disability is constructed [26–28].

Complementing the theoretical constructs in SMD, the socio-ecological perspective draws attention to how the broader social and physical surroundings shape disabilities [28]. Hence this study also draws theoretical insights from the socio-ecological framework, which emphasizes the complex interaction between individuals and their social environments, as well as the multi-layered influence of socio-ecological systems in shaping human development and well-being [25]. The socio-ecological framework holds that microsystems such as family, schools, peer groups, and neighborhoods, form the immediate environment that people interact with. The individual’s interactions with, and the interconnections between these microsystems, external environments (such as the COVID-19 policies that indirectly influence the individual but do not involve the individual directly), and macrosystems (i.e., cultural values, societal norms, economic systems, and political structures) have an overarching influence on their wellbeing [25]. Within the context of the COVID-19 pandemic for instance, macro environmental factors such as the closure of nonessential services, travel bans, lockdowns, and other COVID-19 restrictions have been shown to have mental health ramifications [29–33]*,* thereby highlighting the significant role of the socio-ecological environment in shaping the mental health outcomes of individuals, particularly PWDs who were already confronting everyday challenges. Thus, to understand the unique challenges of PWDs during the COVID-19 pandemic and the impact on their mental health, this study draws its theoretical inspirations from both the SDM and socio-ecological theory.

## Materials and methods

### Ethics statement

Based on the license granted to secondary users of Statistics Canada’s Public Use Microdata Files (PUMF), we required no additional ethical clearance to reuse the 2022 CPSS dataset for analysis and inferences. Kindly visit the following website for details: https://www.statcan.gc.ca/en/microdata/pumf/application/section.

### Data collection and sampling frame

The Canadian Housing Survey (CHS) provides information on how residents of Canada feel about their housing and how housing affects them. CHS data collection covers core housing need, dwelling characteristics and housing tenure, perceptions on economic hardship from housing costs, dwelling and neighbourhood satisfaction, perceptions on neighbourhood issues and safety, housing moves, volunteering, community engagement, life satisfaction, community satisfaction, dwelling adaptations to improve accessibility by PWDs, self-assessed general heath and mental health, experience with homelessness, and sociodemographic characteristics [5].

The 2021 CHS was conducted from January to June 2021 for the provinces and January to March 2021 for the territories using self-response Electronic Questionnaire (rEQ), and Computer Assisted Telephone Interviewing (CATI) as the two main data collection modes. The survey was completed by household members aged 15 years or older with the most knowledge of the household’s housing situation. Proxy response was accepted for questions about other household members. The target population for the 2021 CHS was the population of Canada’s 10 provinces, as well as the territorial capitals of Whitehorse, Yellowknife and Iqaluit. Data collection in the territories was limited to the capitals for operational reasons [5]. The Dwelling Universe File was used as a frame for the 2021 CHS. Administrative data on Social and Affordable Housing (SAH) were used to classify dwellings into strata in the frame. The frame was stratified into geographic areas of interest based on census subdivision (CSD) boundaries. Each geographic stratum was then divided into two groups: SAH dwellings and all other (non-SAH) dwellings. Sub-strata were further used to efficiently sample rented and owned dwellings within the non-SAH strata. Owner occupied dwellings accounted for 68% of the housing stock in Canada. However, given that homeowners have a higher response propensity, the proportion of predicted owners in the CHS sample was 45% of the non-SAH sample. A systematic random sample of dwellings was selected independently within each stratum after sorting by household income, which was obtained from administrative files. For the purposes of the Public Use Microdata File (PUMF), to reduce the risk of disclosure, some of the original CHS geographic strata were grouped. The overall response rate for the 2021 CHS was 47% [5]. All data underlying this study can be found in the PUMF of Statistics Canada via the following link: https://doi.org/10.25318/46250001-eng.

### Measures

The dependent variable for this study is **Positive Mental Health (PMH)**. Participants were asked “In general, how is your mental health?” with “Excellent”, “Very good”, “Good”, “Fair”, and “Poor” as the response categories. Based on earlier studies [3,6,34,35], “Excellent” “Very good” and “Good” were adopted as PMH, the dependent variable for this study. The 2021 CHS dataset was disaggregated to draw data for only individuals with disabilities, for the analysis. The disability variable was a derived variable that indicated the global severity class for respondents (i.e., based on the global severity score, severity classes were established). Severity scores increase with the number of disability types, the level of difficulty associated with the disability, and the frequency of the activity limitation. Therefore, the name assigned to each disability class is only intended to facilitate use of the severity score, and not a label or judgement concerning the person’s level of disability [5]. People in class 1 have a less severe disability than people in class 2. Persons without a disability have an assigned value of 0 for this variable, which should be treated as no disability. Persons with a disability but for whom the severity is unknown (key questions were not answered, making it impossible to derive the score, and hence the class) have a value of 9 (Not stated).

Theoretical relevant independent variables were included in the models for analysis. Namely, Sociodemographic variables: Gender, education, household wealth, immigrant status, household size, household type, presence of racialized minority in household, and experience of economic hardship resulting from COVID-19, civic engagement, dwelling satisfaction, sense of belonging to community. Neighborhood challenges: including whether the neighborhood has problems of race/ethnic-based harassment, vandalism/graffiti/other damage to property, drug use/dealings, drunkenness/rowdiness in public, the feeling of safety in the dark, as well as the problem of noise. Geography: the province of residence was also accounted for in the analysis.

### Analytical approach

Our study is a cross-sectional analysis of the 2021 Canadian Housing Survey (CHS), specifically, Public Use Microdata File (PUMF) from Statistics Canada. Thus, we used descriptive statistics to provide an overview of the sample characteristics. Given the binary nature of the outcome variable, logistic regression models were further employed to examine the association between the independent variables and outcome variable (i.e., Self-rated mental health). Thus, binary logistic regression was conducted at the bivariate level of analysis to understand the relationship between each independent variable and the outcome variable. Furthermore, multiple logistic regression was conducted to evaluate the collective relationships between the independent variables and the outcome variable. The results of the regression models are reported in Odds ratios (OR). Significant Odds ratios above one (OR > 1) indicate a higher likelihood of PMH, while Odds ratios below one (OR < 1) indicate a lower likelihood of PMH. All statistical data analyses were conducted using Stata version 18.

## Results

### Sample characteristics

Out of the 15,626 PWDs (see Table 1), majority (62.22%) were females, had post-secondary education (57.78%), and low SRMH (66.98%). Further, 91.71% of them were Canadian-born citizens/permanent residents, and 8.29% were immigrants. Majority (83.98%) also reported non civic engagement. About 40% reported a weak sense of community belonging. With regards to the province of residence, a larger proportion (20.04%) of them were residents of Ontario. See Table 1 for details on the characteristics of the study sample.

**Table 1:** Descriptive statistics of study sample with disabilities.

Variables	Unweighted frequencies (%)
**Gender**	
Male	5903 (37.78)
Female	9723 (62.22)
**Education**	
High school or lower	6597 (42.22)
Post-secondary	9029 (57.78)
**Wealth**	
Lower	7274 (46.55)
Middle	2702 (17.29)
Upper	5650 (36.16)
**Immigrant status**	
No	14331 (91.71)
Yes	1295 (8.29)
**Household Size**	
1	6905 (44.19)
2	4910 (31.42)
3 and above	3811 (24.39)
**Household type**	
One couple with children	1754 (11.22)
One couple without children	3718 (23.79)
One lone-parent family	926 (5.93)
One census family plus additional person	250 (1.60)
One-person (not in census family)	7136 (45.67)
Two or more persons not in census families	157 (1.00)
Not stated	1685 (10.78)
**Racialized minority in household**	
No	14915(95.45)
Yes	711(4.55)
**Experience Covid-19 economic hardship**	
No	13946 (89.25)
Yes	1680 (10.75)
**Civic engagement**	
No	13122 (83.98)
Yes	2504 (16.02)
**Sense of belonging**	
Weak	6237 (39.91)
Strong	9389 (60.09)
**Dwelling satisfaction due to COVID-19**	
Increased	1202 (7.69)
About the same	12916 (82.66)
Decreased	1508 (9.65)
**Neighborhood issues: harassment base on skin colour/race/ethnicity**	
Not a problem	13226 (84.64)
A problem	2326 (14.89)
Don’t Know	74 (0.47)
**Neighbourhood issues: vandalism/graffiti/ other damage to property**	
Not a problem	11004 (70.42)
A problem	4567 (29.23)
Don’t know	55 (0.35)
**Neighborhood issues: People using/dealing drugs**	
Not a problem	9781 (62.59)
A problem	5762 (36.87)
Don’t know	83 (0.53)
**Neighbourhood issues: People being drunk or rowdy in public**	
Not a problem	11525 (73.76)
A problem	4044 (25.88)
Don’t know	57 (0.36)
**Neighbourhood issues: Feeling safe walking alone in the dark**	
Safe	10153 (64.98)
Unsafe	2548 (16.31)
Don’t walk alone	2925 (18.72)
**Neighbourhood issues: Noise**	
Not a problem	10884 (69.65)
A problem	4728 (30.26)
Don’t know	14 (0.09)
**Region of residence**	
Newfoundland and Labrador	945 (6.05)
Prince Edward Island	576 (3.69)
Nova Scotia	1349 (8.63)
New Brunswick	1531 (9.80)
Quebec	2165 (13.86)
Ontario	3131 (20.04)
Manitoba	1136 (7.27)
Saskatchewan	1177 (7.53)
Alberta	1868 (11.95)
British Columbia	1748 (11.19)

### Bivariate analysis for PMH of PWDs

The results for bivariate analysis are presented in Table 2. Females with disabilities (OR=0.750; P < 0.001) were 0.7 times significantly less likely to report Positive Mental Health (PMH) compared to males. Regarding education, having post-secondary education (OR=1.119; P < 0.001) was significantly 1.12 times more associated with PMH compared to high school or lower level of educational attainment. Also, PWDs in households of racialized immigrants (OR=0.832; P < 0.001) were significantly 0.83 times less likely to report PMH. Compared to single-member households, residents of households with 2 members (OR=1.220; P < 0.001) were significantly 1.22 times more likely to report PMH. Likewise, household types significantly predicted PMH at varied levels (see Table 2). Those who experienced COVID-19 economic hardship (OR=0.575; P < 0.001) significantly reported about 0.58 times less likelihood of PMH. Compared to those with increased dwelling satisfaction, those whose dwelling satisfaction neither improved nor decreased (OR=0.836; P < 0.001), as well as those who reported a decline in dwelling satisfaction (OR=0.420; P < 0.001) was significantly associated with 0.84 and 0.42 times lower odds of PMH, respectively. Also, having a strong sense of community belonging (OR=2.413; P < 0.001) was significantly associated with 2.41 times higher likelihood of PMH compared to a weak sense of community belonging. Residents of neighborhoods with challenges including race/ethnic-based harassments (OR=0.497; P < 0.001), vandalism/graffiti/other damage to property (OR=0.610; P < 0.001), drug use/dealings (OR=0.589; P < 0.001), drunkenness/rowdiness in public (OR=0.548; P < 0.001), feeling of unsafeness in the dark (OR=0.493; P < 0.001) as well as the problem of noise (OR=0.611; P < 0.001) significantly reported lower odds of PMH compared to residents of neighborhoods without such challenges. Furthermore, provincial differences were observed (see Table 2).

**Table 2:** Results for bivariate analysis of PMH.

Variable	Bivariate	
OR(SE)	95% CI
**Gender (**Ref: Male)		
Female	0.750(0.026) ***	0.701, 0.803
**Education (**Ref: High school or lower)		
Post-secondary	1.119(0.038) ***	1.046, 1.198
**Wealth** (Ref: Lower)		
Middle	1.041(0.049)	0.948, 1.142
Upper	0.920(0.034)	0.854, 0.991
**Immigrant status (Ref:** No)		
Yes	1.013(0.062)	0.897, 1.143
Household Size (Ref: 1)		
2	1.220(0.047) ***	1.130, 1.317
3 and above	0.776(0.034) ***	0.711, 0.846
**Household type** (Ref: One couple with children)		
One couple without children	1.833(0.115) ***	1.620, 2.074
One lone-parent family	0.796(0.075) *	0.661, 0.957
One census family plus additional persons	0.790(0.125)	0.578, 1.079
One-person (not in census family)	1.271(0.075) ***	1.132, 1.427
Two or more persons not in census families	0.543(0.118) **	0.354, 0.833
Not stated	1.126(0.084)	0.971, 1.305
**Racialized minority in household** (Ref: No)		
Yes	0.832(0.070) *	0.705, 0.981
**Experience COVID-19 economic hardship** (Ref: No)		
Yes	0.575(0.034) ***	0.510, 0.648
**Civic engagement** (Ref: No)		
Yes	0.258(0.045) ***	0.169, 0.346
**Sense of community belonging (**Ref: Weak)		
Strong	2.413(0.089) ***	2.244, 2.595
**Dwelling satisfaction due to Covid** (Ref: Increased)		
About the same	0.836(0.052) **	0.740, 0.945
Decreased	0.420(0.036) ***	0.354, 0.498
**Neighborhood issue: harassment based on skin colour/race/ethnicity** (Ref: Not a problem)		
A problem	0.497(0.026) ***	0.44, 7.552
Don’t Know	0.781(0.199)	0.474, 1.288
**Neighbourhood issues: vandalism/graffiti/ other damage to property** (Ref: Not a problem)		
A problem	0.610(0.023) ***	0.565, 0.659
Don’t know	1.012(0.284)	0.583, 1.756
**Neighborhood issue: People using/dealing drugs** (Ref: Not a problem)		
A problem	0.589(0.021) ***	0.548, 0.633
Don’t know	0.859(0.200)	0.544, 1.356
**Neighbourhood issues: drunkenness or rowdiness in public** (Ref: Not a problem)		
A problem	0.548(0.022) ***	0.505, 0.595
Don’t know	0.746(0.216)	0.422, 1.318
**Neighbourhood issues: feeling of safeness walking alone in dark** (Ref: Safe)		
Unsafe	0.493(0.025) ***	0.445, 0.546
Don’t walk alone	0.903(0.040) *	0.828, 0.985
**Neighbourhood issues: Noise** (Ref: Not a problem)		
A problem	0.611(0.023) ***	0.566, 0.659
Don’t Know	0.980(0.547)	0.328, 2.927
**Region of residence (Ref:** Newfoundland and Labrador)		
Prince Edward Island	0.706(0.078) **	0.568, 0.878
Nova Scotia	0.722(0.063) ***	0.608, 0.859
New Brunswick	0.739(0.063) ***	0.625, 0.874
Quebec	1.111(0.088)	0.951, 1.298
Ontario	0.590(0.045) ***	0.507, 0.686
Manitoba	0.721(0.066) ***	0.602, 0.863
Saskatchewan	0.731(0.066) ***	0.612, 0.874
Alberta	0.623(0.052) ***	0.529, 0.734
British Columbia	0.649(0.054) ***	0.550, 0.766

*P < 0.05; **P < 0.01; ***P < 0.001; Odd Ratio (OR), Standard Error (SE), Confidence Interval (CI), Number of observations = 15,626.

### Multivariate analysis for PMH of PWDs

The results for multivariate analysis are presented in Table 3. Consistent with results at the bivariate level, being a female (OR=0.789; P < 0.001), decreased dwelling satisfaction (OR=0.585; P < 0.001), as well as the experience of COVID-19 economic hardship (OR=0.703; P < 0.001) was significantly associated with 0.79, 0.59, and 0.70 times lower odds of PMH, respectively. On the other hand, being an immigrant (OR=1.204; P < 0.05), having post-secondary education (OR=1.210; P < 0.001), a strong sense of community belonging (OR=2.056; P < 0.001), and civic engagement (OR=1.204; P < 0.001) were significantly associated with 1.20, 1.21, 2.06, and 1.20 times higher odds of PMH, respectively. Regarding household type, compared to one-couple households with children, PWDs from one-couple households without children (OR=1.543; P < 0.001) significantly reported 1.54 times higher likelihood of PMH. However, residents of neighborhoods with challenges such as race/ethnic-based harassments (OR=0.790; P < 0.001), drug use/dealings (OR=0.882; P < 0.05), drunkenness/rowdiness in public (OR=0.867; P < 0.05), feeling of unsafeness at night (OR=0.794; P < 0.001), as well as noise (OR=0.863; P < 0.001), all significantly reported lower odds of PMH compared to residents of neighborhoods without such challenges. Provincial differences were further observed (see Table 3).

**Table 3:** Results for multivariate analysis of PMH.

Variable	Multivariate	
	OR(SE)	95% CI
**Gender (**Ref: Male)		
Female	0.789(0.030) ***	0.731, 0.850
**Education (**Ref: High school or lower)		
Post-secondary	1.210(0.047) ***	1.121, 1.306
**Wealth** (Ref: Lower)		
Middle	0.989(0.054)	0.888, 1.101
Upper	0.927(0.047)	0.839, 1.024
**Immigrant status (Ref:** No)		
Yes	1.204(0.093) *	1.034, 1.402
**Household Size** (Ref: 1)		
2	0.937(0.172)	0.653, 1.345
3 and above	0.901(0.141)	0.663, 1.225
**Household type** (Ref: One couple with children)		
One couple without children	1.543(0.183) ***	1.223, 1.947
One lone-parent family	0.917(0.108)	0.726, 1.157
One census family plus additional persons	0.829(0.136)	0.601, 1.144
One-person (not in census family)	1.170(0.193)	0.847, 1.618
Two or more persons not in census families	0.601(0.144) *	0.375, 0.964
Not stated	1.272(0.106) **	1.079, 1.500
**Racialized minority in household** (Ref: No)		
Yes	1.044(0.109)	0.851, 1.282
**Experience COVID-19 economic hardship** (Ref: No)		
Yes	0.703(0.044) ***	0.621, 0.796
**Civic engagement** (Ref: No)		
Yes	1.204(0.057) ***	1.096, 1.323
**Sense of community belonging (**Ref: Weak)		
Strong	2.056(0.080) ***	1.904, 2.220
**Dwelling satisfaction due to Covid** (Ref: Increased)		
About the same	0.850(0.055) *	0.748, 0.967
Decreased	0.585(0.053) ***	0.489, 0.700
**Neighborhood issue: harassment based on skin colour/race/ethnicity** (Ref: Not a problem)		
A problem	0.790(0.050) ***	0.696, 0.896
Don’t Know	1.021(0.295)	0.579, 1.799
**Neighbourhood issues: vandalism/graffiti/ other damage to property** (Ref: Not a problem)		
A problem	0.924(0.045)	0.840, 1.017
Don’t know	1.376(0.419)	0.757, 2.502
**Neighborhood issue: People using/dealing drugs** (Ref: Not a problem)		
A problem	0.882(0.041) **	0.804, 0.968
Don’t know	1.055(0.282)	0.624, 1.784
**Neighbourhood issues: drunkenness or rowdiness in public** (Ref: Not a problem)		
A problem	0.867(0.048) *	0.778, 0.967
Don’t know	0.770(0.257)	0.400, 1.482
**Neighbourhood issues: feeling of safeness walking alone in dark** (Ref: Safe)		
Unsafe	0.794(0.046) ***	0.709, 0.890
Don’t walk alone	1.048(0.051)	0.952, 1.155
**Neighbourhood issues: Noise** (Ref: Not a problem)		
A problem	0.863(0.038) ***	0.790, 0.941
Don’t Know	0.724(0.431)	0.225, 2.327
**Region of residence (Ref:** Newfoundland and Labrador)		
Prince Edward Island	0.680(0.078) ***	0.543, 0.852
Nova Scotia	0.718(0.065) ***	0.600, 0.859
New Brunswick	0.739(0.065) ***	0.621, 0.880
Quebec	1.207(0.100) *	1.026, 1.420
Ontario	0.664(0.054) ***	0.566, 0.780
Manitoba	0.810(0.077) *	0.671, 0.977
Saskatchewan	0.788(0.074) *	0.654, 0.949
Alberta	0.755(0.066) ***	0.636, 0.897
British Columbia	0.724(0.065) ***	0.607, 0.864

*P < 0.05; **P < 0.01; ***P < 0.001; Odd Ratio (OR), Standard Error (SE), Confidence Interval (CI), Number of observations = 15,626.

## Discussion

Guided by the social disability model (SDM) and socio-ecological framework, this study explored the factors that influenced the mental health of persons with disabilities (PWDs) during the COVID-19 pandemic in Canada. PWDs often face additional health risks or vulnerabilities, and the fear of contracting COVID-19 during the pandemic may have heightened these risks, contributing to increased stress and anxiety. Also, PWDs, particularly, physically disabled individuals rely on support services such as in-person healthcare, personal assistance, and other community support programs. The disruption or limitation of these services during the COVID-19 pandemic led to feelings of helplessness and isolation, loss of routine, all of which contributed to heightened MH challenges. Social isolation is a major risk factor for mental health issues, and the pandemic’s restrictions undoubtedly exacerbated feelings of loneliness. The uncertainty surrounding the duration and impact of the pandemic, coupled with housing suitability concerns and the availability of healthcare resources, have particularly contributed to heightened anxieties among PWDs. Our study results showed that being a female, experience of COVID-19 related economic hardship, decline in dwelling satisfaction, as well as residents in neighborhoods with challenges such as race/skin/ethnic-based harassment, vandalism/graffiti, noise, feeling of unsafeness at night, and drug use/dealings, were significantly associated with lower odds of reporting PMH. On the other hand, being an immigrant, belonging to one-couple household without children, post-secondary educational attainment, civic engagement with the local community, and having a strong sense of community belonging were all factors significantly associated with higher odds of reporting PMH among PWDs. Moreover, the province of resident was a significant predictor of PMH in Canada. These findings are discussed in the ensuing paragraphs.

Gendered disparities were observed as females with disabilities reported lower odds of PMH relative to their male counterparts. The prevalence of depressive symptoms and anxiety disorders in Canada between the year 2020 and 2021 were reportedly more pronounced among females [1]. Also, aside Statistics Canada’s reports showing that women of all ages in Canada are more likely than men to have a disability [20], reports from the Public Health Agency of Canada (PHAC) [36] further indicated that self-reported signs and symptoms of mental health often differ between men and women—where women tend to report more feelings of helplessness than men. An earlier study had unpacked how females in vulnerable positions, including expectant and postpartum mothers and those who experienced intimate partner violence were at higher risk of poor MH during the pandemic [37]. Other females such as the employed, the married, and single parents experienced a deteriorating MH due to disproportionately shouldering of caregiving responsibilities and household duties during the pandemic [37]. For instance, a study by Zamarro & Prados [38] on gender differences in couples’ division of childcare, work and mental health during COVID-19, showed that women bear the brunt of childcare provision than men during the pandemic. The study further observed a gap in psychological distress that emerged between mothers and women without children of school-going age in the household during the pandemic [38]. The COVID-19 pandemic may have amplified these pre-existing gender inequalities, especially among PWDs who were already facing socio-environmental impairments in performing their daily tasks and responsibilities. These stressors, coupled with social isolation and heightened concerns about health and safety, can lead to poor mental health outcomes. It is however worthy to highlight that in addition to reporting higher levels of distress than males, females are also more likely than males to seek help from healthcare professionals for their MH concerns [36]. Masculinity and/or societal expectation of men to be tough and strong is a major barrier that prevent most men from seeking help. Thus, while it is important to address the special needs of women with disabilities, concerted efforts should be made to encourage the reporting of mental health challenges, and use of mental health services by men.

Regarding household types, households consisting of one couple without children reported higher odds of PMH compared to one couple households with children, a finding that aligns with that of prior studies [38,39]. The absence of childcare responsibilities can offer childless couples greater flexibility and autonomy, ultimately relieving them from parenting stress. Without the added demands of homeschooling or childcare, and concerns about children’s well-being, PWDs may have more time for personal well-being and self-care, contributing to a more positive mental health assessment. In contrast, those who have children may face increased stressors, including managing their children’s education, childcare, and the overall well-being of the family unit [38]. Balancing work commitments with parenting responsibilities during lockdowns and school closures can lead to higher levels of stress with negative impacts on mental health. This, therefore, underscores the need to prioritize addressing the needs of PWDs who has childcare responsibilities, especially during a public health crisis.

Furthermore, post-secondary educational attainment, and being an immigrant were associated with higher odds of PMH. This observation may be due to several factors, including the likelihood of greater help-seeking behavior, diminishing stigma, or increasing mental health literacy among people with higher educational qualifications. Also, individuals with higher qualification in Canada may be better equipped with critical thinking skills, and exposed to a broader range of resources that they can rely on during critical periods like the COVID-19 pandemic and related uncertainties and challenges. For instance, a nationwide Canadian-based study [40] revealed that some post-secondary institutions in Canada offer student-to-student or peer health educator programs that instill students with stress-coping skills which positively impact their mental health/well-being. A more recent study by Wiens et al. [41] further establishes that nonstudents are more likely to experience poor mental health outcomes compared to postsecondary students. The positive association between being an immigrant and having PMH in our study context is rather surprising given that prior studies have emphasized that the diverse religious and cultural backgrounds and complex mental health-related concerns of immigrants and refugees are not well integrated into existing mental health services in Canada [42,43]. The underutilization of mental health services by immigrants stems from a multiplicity of factors, including lack of knowledge of existing health information and services, barriers related to the availability of appropriate services, and the compounding challenges of resettlement and integration faced by immigrants [42]. Socio-cultural, ethno-religious, linguistic, and economic barriers are notable barriers being faced by immigrants in Canada with regards to mental health service utilization [42–45]. Despite these, we posit that our findings could potentially be reflecting the “healthy immigrant effect” where immigrants enjoy better health than native-born people albeit they experience a decline in health over time in their destination areas [46–48]. Additionally, other studies also highlighted that immigrants often bring unique qualities such as resilience developed through their migration and settlement experiences [49], as well as cultural coping mechanisms that can contribute to their adaptability [50,51]. It is possible that in the context of our findings, these cultural coping skills in Canada among immigrants, may have mitigated the impact of pandemic-related stressors on the mental well-being of foreign-born PWDs in our study context.

The results further shows that PWDs who experienced COVID-19 related economic hardship, reported lower odds of PMH. Scholarly studies suggest that PWDs are more vulnerable than others to financial hardship and low social support, both of which are linked with poor mental health outcomes [52]. They also face unequal job opportunities [53], and even when employed, they face discriminations based on their disability status [54]. Consequently, PWDs are shown to have limited sources of income or lower earnings relative to those without any physical or psychological impairment [55]. Thus, those who experienced COVID-19 induced economic hardships would most likely struggle to meet basic needs, pay bills, or support their families. For example, economic hardship may lead to challenges maintaining housing, including rent or mortgage payments. The fear of eviction can heighten a sense of instability and anxiety from the pandemic and ultimately impact mental health. Besides, economic hardship can limit an individual’s ability to maintain their previous lifestyle and engage in leisure or stress-relieving activities such as exercise, hobbies, or socializing. Disruptions to these daily life activities can negatively affect overall well-being, exacerbate feelings of frustration, helplessness, and decreased life satisfaction. This finding reaffirms prior studies that have evaluated the impacts of economic hardship on mental health and well-being [56–58]. The finding thus underscores the importance of prioritizing the provision of financial relief packages for PWDs who may be unemployed or from low socio-economic backgrounds, especially during critical times like the COVID-19 pandemic.

The significant association between civic engagement and PMH in our study context aligns with prior studies that highlight the net effect of civic engagement in reducing depressive symptoms of populations and improving mental health and well-being [59]. Civic engagements, such as volunteering or participating in community initiatives, provide individuals with a sense of purpose and contribution. Immersing oneself in meaningful and purposeful activities can redirect focus away from negative thoughts, contributing to improved mental well-being [59]. Given that civic engagement often involves some interaction with others who share similar interests and values, it can serve as a vital support network, reducing feelings of social isolation that may have been caused by pandemic-related restrictions [60]. Also, civic engagement can contribute to a sense of belonging within a community, and the feeling of connectedness and integration into one’s community has positive implications for mental health [61], especially during challenging times.

A strong sense of community belonging provides PWDs a social support network. Particularly, during the pandemic where physical distancing measures and lockdowns led to widespread social isolation, individuals with strong sense of community belonging may still benefit from their connectedness that provides them emotional support [62]. Sense of community belonging often involves sharing common values, norms, and experiences. During the pandemic, it is possible that individuals with a strong sense of community belonging may have found comfort and understanding among others who share similar perspectives, which can positively impact mental well-being. A strong attachment to one’s community may also foster a sense of collective efficacy where individuals believe in their community’s ability to work together to overcome challenges. These shared beliefs in the community’s ability to address problems can enhance individuals’ confidence and reduce feelings of helplessness during the pandemic. On the contrary, weaker sense of community belonging is shown to be associated with both poorer general health and mental health [63]. Overall, a sense of community belonging creates a psychologically safe environment, provides a safety net that can reduce stress and anxiety, and fostering positive mental health environment. This therefore underscores the crucial need to create more opportunities for civic engagement of PWDs in Canada.

Moreover, given that the COVID-19 pandemic led to increased reliance on homes as safe havens due to lockdowns and social distancing measures, it is not surprising that individuals who experienced a decline in dwelling satisfaction were significantly associated with poor mental health in this study. Within the context of housing conditions and COVID-19 outbreak, a recent study discovered that the level of housing satisfaction has significant influence on the mental health of occupants [64]. The decline in dwelling satisfaction stemming from COVID-19 may result in feelings of discomfort (i.e., limited space or inadequate living conditions), potentially contributing to increased stress and anxiety. Spending more time at home during the pandemic could amplify the negative impact of these factors on mental well-being. As a housing characteristic, limited access to nature and fresh air can contribute to feelings of confinement and negatively impact mental health.

Neighborhood challenges, including race/ethnic-based harassment, drug use/dealing, the feeling of unsafeness at night, and noise, were significantly associated with lower odds of positive mental health. A community-based study in Los Angeles County indicated that the perception of people regarding their neighborhood influences their mental health—the more threatening the neighborhood, the more prevalent the symptoms of depression, anxiety, and conduct disorder [65]. Thus, persistent exposure to race/ethnic-based harassment, vandalism, and drug-related activities in residential areas can create a heightened sense of stress and anxiety among residents, especially during uncertain times like the COVID-19 pandemic. For instance, a more recent study has found a positive association between experience of racial discrimination and a higher likelihood of psychological distress [66]. Likewise, perceived feeling of insecurity is determinantal to mental health [67]. Residents of neighborhoods facing such challenges may also experience a perceived lack of control over their living environment, and the inability of individuals to address such issues can lead to a sense of helplessness and contribute to poorer mental health outcomes. This underscores the urgent need for policymakers to identify and address neighborhood problems as part of an effort to combat the mental health crisis in the country. Provincial differences were further observed. For instance, aside from Quebec, PWDs from all the other provinces were significantly associated with lower odds of PMH, relative to the residents of Newfoundland and Labrador. A nationwide study of Canada’s provincial and territorial differences on COVID-19 response have shown that Newfoundland and Labrador experienced more freedom and ‘normalcy’ during the pandemic than the larger and more populous central and western provinces [68], a factor that may have reduced the level of anxieties and depression that accompanied the COVID-19 restrictions. Also, variations in the availability of readily accessible and affordable mental health support services and COVID-19 relief funds, especially for PWDs may have also played a role in the observed association. Overall, the study unveiled the differential experiences of the pandemic’s impact on the mental health of PWDs, as well as their unique vulnerabilities to public health crises. Particularly, women, the less educated, and those from weak socioeconomic backgrounds living with disabilities appeared to be the most vulnerable in our study context.

### Study limitations

Although this study adds valuable insights to the existing literature on disability and mental health during the pandemic, some limitations are worth highlighting. First, the quantitative nature of the study limits the interpretation of the findings to statistical association. Additionally, the self-reported nature of the measure of mental health makes it susceptible to response bias. More so, our study is not exhaustive of all the measures of PMH as conceptualized in other studies [11]. That notwithstanding, the study presents significant insights from the COVID-19 pandemic, relevant for policy integration and planning for PWDs in future outbreaks.

## Conclusions and recommendations

Employing the lens of social disability model (SDM), we conclude that recognizing and addressing the unique MH needs of persons with disabilities (PWDs), especially during public health crisis, is very crucial. This may include looking at the intersection of gender disparities, economic and dwelling conditions, and sense of belonging among other things, which have emerged to be crucial for promoting PMH among PWDs during the COVID-19 pandemic. The findings of this study also have several policy implications. First and foremost, the observed lower odds of PMH among women relative to men suggest that policy interventions (i.e., both new and existing) seeking to address the mental health challenges of PWDs in Canada must prioritize identifying and integrating the unique challenges of women, especially the less educated and those facing economic hardship. This could involve financial assistance programs, mental health resources, and targeted outreach to these populations. Access to educational opportunities must also be expanded among PWDs given the positive impact of higher education on mental well-being. Policies should prioritize scholarships, mentorship programs, and support services for persons with disabilities. Secondly, there must be deliberate policy efforts to enhance a strong sense of community belonging among PWDs. Programs that foster a sense of community belonging and civic engagement can improve mental health outcomes. Thus, local governments and organizations should invest in community-building initiatives that promote social connections or the inclusivity of PWDs. It is equally imperative to address neighborhood-specific challenges—such as race-based harassment and substance abuse—through integrated social services that can provide comprehensive support for individuals, enhancing overall mental health. Last but not the least, data-driven decision making, and inclusive policy development is undoubtedly crucial to promoting positive mental health among PWDs. Therefore, there must be continued research and data collection on mental health trends among PWDs to inform effective policy responses and resource allocation. In doing so, individuals with disabilities must be involved in the development of mental health policies to ensure their perspectives and needs are adequately represented and addressed.
